# Operators’ Load Monitoring and Management

**DOI:** 10.3390/s20174665

**Published:** 2020-08-19

**Authors:** Utku Kale, József Rohács, Dániel Rohács

**Affiliations:** Department of Aeronautics, Naval Architecture and Railway Vehicles, Faculty of Transportation Engineering and Vehicle Engineering, Budapest University of Technology and Economics, H-1111 Budapest, Hungary; jrohacs@vrht.bme.hu (J.R.); drohacs@vrht.bme.hu (D.R.)

**Keywords:** operators, automation, passive monitoring, operator loads, load management

## Abstract

Due to the introduction of highly automated vehicles and systems, the tasks of operators (drivers, pilots, air traffic controllers, production process managers) are in transition from “active control” to “passive monitoring” and “supervising”. As a result of this transition, the roles of task load and workload are decreasing while the role of the mental load is increasing, thereby the new type of loads might be defined as information load and communication load. This paper deals with operators’ load monitoring and management in highly automated systems. This research (i) introduces the changes in the role of operators and requirements in load management, (ii) defines the operators’ models, (iii) describes the possible application of sensors and their integration into the working environment of operators, and (iv) develops the load observation and management concept. There are some examples of analyses of measurements and the concept of validation is discussed. This paper mainly deals with operators, particularly pilots and air traffic controllers (ATCOs).

## 1. Introduction

The transportation sector is expected to continue growing considerably over the next two decades. In dealing with this growth, it is essential to ensure the highest level of safety and security. The revolution in information, computer, navigation and communication technologies catalyses the development of highly automated systems in operators’ environments, such as future transport systems. In parallel to these rapid technological changes, a large number of companies, universities, and institutes have initiated intensive research on the future of autonomous systems. Worldwide, several mega international and national projects have been initiated for the research, development, and implementation of systems, regulations, and procedures for future transportation systems, such as Vision Zero [1], eSafety [2], White Paper [3], European SESAR—Single European Sky Air Traffic Management (ATM) Research [4], US Next-Gen—Next Generation Air Transportation System [5], Japanese CARATS—Collaborative Actions for Renovation of Air Traffic System [6], and Brazilian SIRIUS—Impulsionando o Desenvolvimento do ATM Nacional [7]. One of their main objectives is to bring an extensive range of innovative solutions to the current transportation problems and as well as to cope with future problems. These investigations have been introduced to countless technological and system innovations in the working environment of operators. Thereby, the level of automation, vehicle systems and the working conditions of the operators are continuously changing and being redesigned. Transportation operations, therefore, must continue to adapt accordingly and meet the needs of the growing transportation industry.

In a continually changing world, the role of operators (drivers, pilots, air traffic controllers (ATCOs)) is in a transition from active endogenous control to passive monitoring due to the introduction of the intensive automation. It means that, before, operators were actively taking part in the controlling process, however now they are in a position of monitoring the current system. In the case of active control, the operator takes control, depending on several factors such as skills, experience, and tacit knowledge, etc. However, in the case of passive monitoring, operators become observers rather than controllers of vehicles. These transitions also introduce several vital changes in the operators’ work and more vehicle-related problems into the system. As the level of automation was increased, the human factors, operator loads, situation awareness and decision-making processes became more critical. In this sense, operators need viable constructs, principles and transport systems to promote a better understanding of automation and balancing their loads in complex systems.

### 1.1. Motivation

The complex and dynamic environment measures of operator performance are more complicated than during the early stage of transportation. Despite all the advancements in transport technology and automation, accidents continue to occur. With the continuous evolution of transport systems, operators are supported by vast amounts of available data and relevant information. Too much available information confuses operators during operation, particularly during the decision-making process in abnormal/emergency situations. In this system, information and communication load were detected as new types of operator loads. In addition to this, the role of mental condition is increased, and, tasks and workload become more interconnected. In the modern operator environment, the role of the operator has become an information manager rather than an operator. This highly automated system may be accompanied by unbalanced operator load systems (varying from under load to overload), unintended reductions in situation awareness, decreases in the quality of decision-making, and increased levels of stress.

### 1.2. Contribution

In this paper, a new operator load model was created and the operator load divided into five categories, namely work, task, information, communication and mental load. In addition to this, the “Swiss Cheese model”, “load model” and “information model” were improved and adapted to human operator work. The main objective of this research is to develop general load monitoring and management systems for operators working in highly automated systems. Advances in sensor and data integration technologies in the current transport systems, allow researchers to collect, measure, monitor, and evaluate data prospectively with innovative devices. Such applications like video camera, electrodermal activity (EDA) devices, eye-tracking device and outside measuring equipment were used in the flight simulator and Air Traffic Control (ATC)/ATM simulation laboratory of the Department of Aeronautics, Naval Architecture and Railway Vehicles (VRHT) at Budapest University of Technology and Economics (BME). The overall result of this study shows that operator total load systems can be monitored by integrated sensors in real-time and managed by the developed methodology. These improvements, (i) help operators to balance their total loads during operation, (ii) improve the operator’s situation awareness and decision-making process, (iii) let instructors better understand the weaknesses of operators, and (iv) improve the quality of operator training.

## 2. Materials and Methods

### 2.1. Influence of Automation on Loads of Operators

While technology has helped drive improvements in the transportation industry, automation has also increased significantly and is going to go on advancing at an increasing rate. Automation in transport aims to improve safety outcomes by reducing human errors and improving the comfort of operators (personal productivity and pleasure). Automation affects all modes of transportation including rail, maritime, air and road transport while changing the vehicles, operation systems, and operator working environments. The modern vehicles use smart sensors and cameras to assist drivers and eliminate human errors. In current technology, transport systems are capable of carrying out many tasks that need to be performed by operators. According to Parasuraman, automation is a device or system that accomplishes (fully or partially) a function that was previously carried out (fully or partially) by the human operator [8]. Automation in transportation can be defined as an innovative and modernised technology to monitor and control devices or systems to reduce the need for operator intervention and activities. In vehicles, most of the driver tasks rely on automation, whether she/he wishes to perform a task by automation.

**Figure 1:**
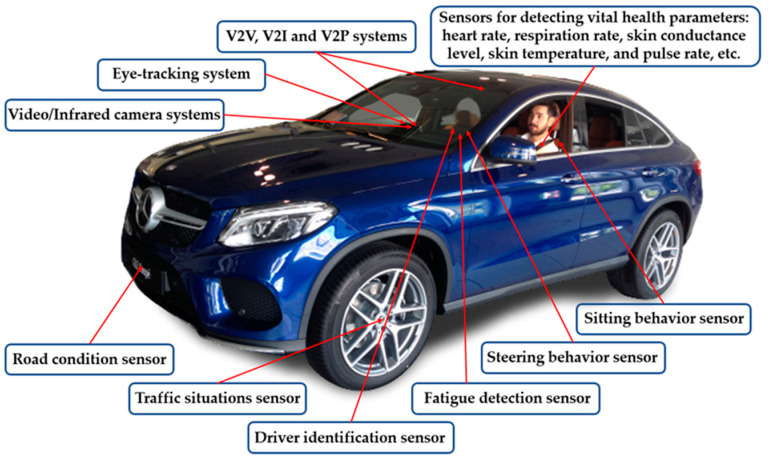
Futuristic in-car technologies.

Undoubtedly, automation brought countless advantages to transport systems and solved many past and current problems such as traffic collisions, accidents, congestion, and environmental pollution, etc. However, technological advances in automation and computing processes have produced highly complex driver–machine systems. One of the drawbacks of automation is that the role of the operator continues being affected by automation. The role of vehicle drivers and transport controllers is transitioning from active control to passive observing and supervising with control systems. The passive operator role at a high level of automation creates unbalanced total load systems, particularly in abnormal/emergency situations. Operators, therefore, need viable constructs, principles and transport systems to promote a better understanding of automation and balancing their loads in complex circumstances. In this research, total load monitoring systems were integrated and tested in pilots’ and ATCOs’ working environments. However, the developed load management methods and sensor technologies can be applied not only in cockpits and ATMs but also in the working environment of all other modes of transportation (air, road, rail, maritime) [9,10,11,12]. Figure 1 shows futuristic in-car technologies as an example. With the help of the advanced sensors and cameras, modern cars can monitor and store information about the driver’s vital health parameters, traffic situation, road condition, and the driver’s ability and limitations. This measurable information can be used to identify a total load on drivers to assist operators and control the vehicle when it is required such as in an unforeseen/emergency situation.

The first signs of aircraft automation were introduced on-board aircraft during the decade from 1920 to 1930, in the form of an autopilot based on a mechanical engineering concept that was designed to keep the aircraft flying straight [13]. In the 1960′s warning and alerts, systems were developed, and in the 1970s, engine fire warning systems were installed in the Boeing 707, Boeing 747, and Boeing 777 [14]. From the 1970s to the present day, there have been countless innovations introduced on-board aircraft that enhance safety: electronic autopilots, auto-throttle, flight directors, airborne weather radars, navigational instruments, and inertial platforms [13].

Air traffic control is a real-time safety, critical decision-making process that is highly dynamic. The “Air Traffic Management System”, as it is used today, is centrally controlled. The modern workstation is designed to integrate several distinct subsystems into one system [5,15,16]. In designing the working environment of ATCOs, it is necessary to take into consideration not only physical factors like the positioning of radar screens, lighting, auxiliary and visual displays but also subjective factors such as skill, ability, experience, loads, anxiety, and stress. Further standardization of the panel layout is required as well. All controls in a cockpit should be within easy reach of the crew, and all instruments should be easy to read and understand by operators. The performance of the overall pilots and flight deck system depends on understanding the total system. This situation will permit pilots to acquire information without interference and allow them to operate all the controls efficiently for effective, environmentally friendly and safe operation [17,18,19,20].

Concerning the working environment of pilots, aircraft cockpits and its systems have evolved considerably over the decades. Past flight deck design practices have been highly successful in producing safe and efficient aircraft. Technological advances provide improvements in pilots’ working environments and will continue to do so in the future. The level of automation has changed the way pilots control aircraft and their needs in the working environment. Due to these changes, flight deck design needs to be reconsidered accordingly. The position and operation of controls and flight instruments are crucial for operators. Pilots have to carry out their tasks based on the information given on instrumental panels, and being received from ATCOs and other pilots. Advances in sensor and data integration technologies in aviation systems make more information available than ever before. On the other hand, highly automated systems have increased the monitoring tasks of pilots instead of reducing control tasks. This phenomenon will cause pilots to lose their “situation awareness” during flight operations.

**Figure 2:**
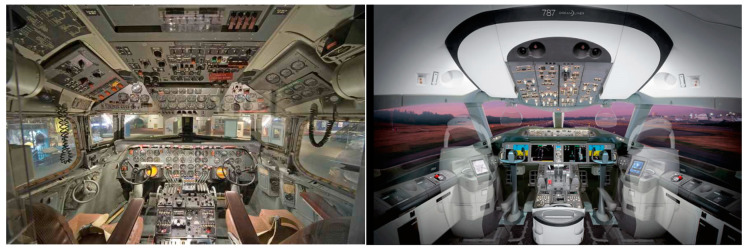
Flight deck of a Douglas DC-7 (left) [21] and Boeing 787-9 (right) [22].

A comparison example of cockpit designs in the earlier days of aviation and the modern cockpit environment is shown in Figure 2. As seen in this figure, the cockpit design has been changed significantly with increasing display areas and a smaller number of individual displays. In the earlier days of aviation, analogue instruments were used for information presentation since the 1960s [23]. With the introduction of new avionics systems and increasing levels of automation, in the early 1970s Multi-Functional Displays (MFD) appeared in the cockpit to display information to the operators in numerous configurable ways while a gauge can only display a limited amount of data. With the advent of microprocessors, microelectronics, and Liquid Crystal Display (LCD) technology in the 1980s, the first MFDs based on LCDs with their computer within the display appeared in the aircraft cockpit [24]. In the early days of aviation, researchers and aircraft designers introduced as many more gauges to aircraft to be able to provide reasonable information to pilots for the proper and safe performance of a flight. In cockpits designed earlier, gauges could only display a limited amount of data. However, after rapid changes in the level of automation, cockpits are designed to decrease the number of small instruments. At first sight, the cockpit as a working environment has significantly simplified for the last two decades. Unfortunately, the new cockpit instrumentation introduced some new problems too such as:

- designers must find the optimal examining solutions for indicating the measurements and information, namely presentation in analogue or digital forms,
- the applied display may show too many and rather different information during a short time,
- new, highly automated cockpit instrumentation and the working environment of pilots increase the role of the mental load and brings in new types of load, information and communication overload.

With continuous evolution of the flight decks, including aircraft capabilities and sensor systems pilots, are supported by huge amounts of available data and relevant information. In the current modern cockpit environment, the role of pilots has become an information manager rather than an operator. The huge amount of available information confuses pilots during operation, particularly while decision-making in abnormal/emergency situations. While the introduction of new technology brings significant improvements and may solve some problems [25,26,27,28], it often introduces others in all transportation modes [29,30,31,32]. The future working environment of pilots (cockpit and future ground control towers) needs to be designed by taking into account various psychological and human factors.

This paper comprises of the evaluation, monitoring, and management of the total load systems of operators, considering their current needs. In that context, load monitoring devices were created and performed measurements in the working environment of operators. The developed systems can be used in the working environment of operators in order to balance “ total load”, increase “situation awareness” and improve the “quality of decisions”.

### 2.2. Operator Models

The operator model can be defined by three different approaches: (i) Swiss Cheese model, (ii) load model, and (iii) information processing model.

(i) Swiss Cheese Model: Operator errors during the decision-making process might be due to such factors as under/overload, stress, fatigue, available time, and lack of tacit knowledge, etc. According to James Reason [33], human errors can be divided into two categories: the person approach (errors of individuals) and the system approach (working condition of humans). James Reason proposed the model of “Swiss Cheese” to explain the occurrence of system failure [34]. In this model, accidents and incidents are caused by a set of errors in complex circumstances. Each layer has several holes that represent individual weaknesses in individual parts of the system—hence the similarity to slices of randomly-holed Swiss Cheese. These holes are arranged vertically and parallel to each other with gaps in-between each slice and are continuously opening, closing, and changing their location depending on the current situation in complex systems. When by chance, all holes are aligned, this can cause unfortunate outcomes such as accident and incident.

**Figure 3:**
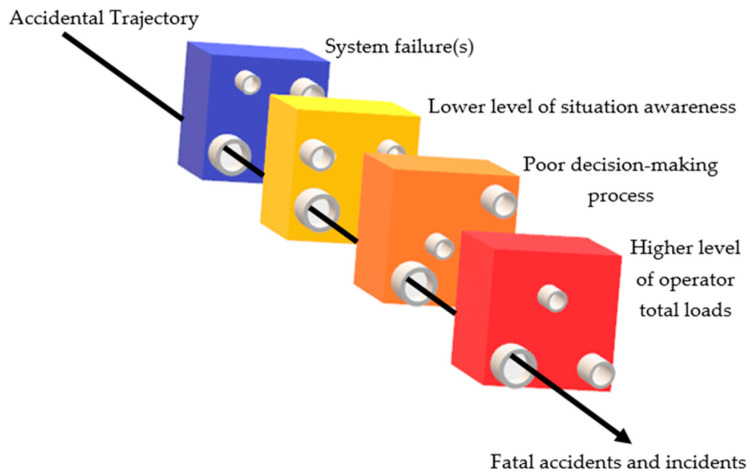
The Swiss Cheese Model (SCM) of operator error causation.

**Figure 4:**
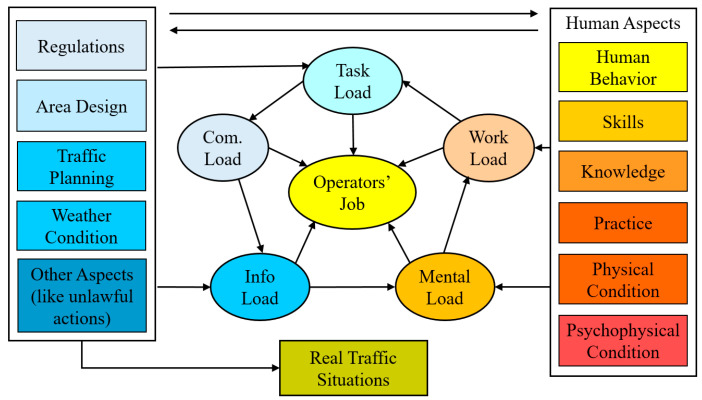
The improved “Operator Load Model” published by Endsley [41].

Figure 3 shows the “Swiss Cheese Model” of how an accident trajectory may penetrate a vehicle accident. The vehicle accident might occur by way of multiple human errors or system failures in each level in the system line up that influence each other (passes through holes in all of the defences) such as loss of situation awareness, distractions, lack of experience and inadequate training.

(ii) Operator Load Model: Another approach can be applied to the description of the operator model based on the operator load. One of the well-known load models is described by Endsley. However, with the changes in the role of operators from active control to passive monitoring, there is a need for redesigning of the Endsley load model. With rapid technological changes, in many cases, information, communication and mental load became potential problems that require aviation systems to monitor and control them. These load systems, therefore, were defined separately in the new model (Figure 4). In this way, operator load is divided into five groups, namely work, task, information communication, and mental load, which called operator total load. The operator total load monitoring and management systems were created and tested in a flight simulator.

- The task load indicates the degree of difficulty and hardness when executing a task such as traffic regulations, traffic demands, area design and other task demands. In the case of highly automated systems, changes in traffic intensity and abnormal and emergency situations may generate several extra tasks. This load can be assessed by the “NASA Task Load Index” (NASA-TLX) [35].
- The workload is defined as the total amount of work by an operator in a specific time period as it depends on both human factors (human behaviour, skill, tacit knowledge, experience, etc.) and external factors (traffic regulation, traffic planning, weather condition, etc.). Several techniques have been proposed for measuring and evaluating operator loads. The most well-known measuring techniques are the following: (i) “Situation Awareness Global Assessment Technique” (SAGAT) [36], (ii) “Situation Awareness Rating Technique” (SART) [37], (iii) “Automation Thrust Index” (SATI) [38], (iv) Subjective Workload Assessment Technique (SWAT) [39], and (v) Workload Profile (WP) [40].
- As an operator workplace environment becomes increasingly complex, the amount of information gained from vehicle systems is also increased. The automated vehicle systems provide too much information to operators from different sources. The amount of information creates confusion among operators. Information load, therefore, was created and included in the Endsley load model. This load depends on traffic regulation, weather condition and other aspects.
- Communication load can be defined by the level of understanding between operators which depends on language, cultural norms, and social relations. This load, therefore, is included in the Endsley load model as well (Figure 4).
- Mental load is the physical and psycho-physiological condition of operators and strongly depends on human aspects (behaviours, skills, knowledge, experience, physical and psychophysical condition). The quality of situation awareness and decision-making process is directly affected by the mental load of operators. In the literature, numerous articles have been published in the field of mental load [42,43]. The mental load of operators is measured by using medical and physiological devices.

**Figure 5:**
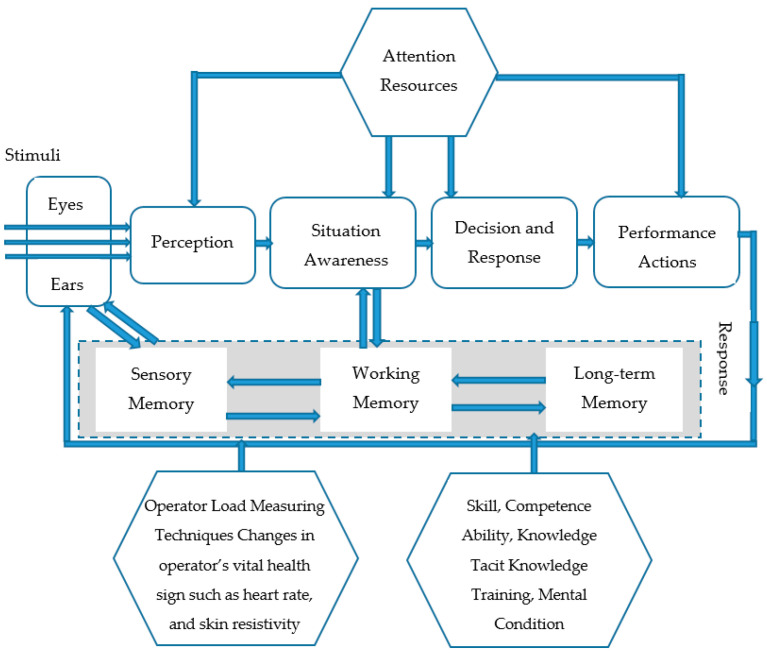
The improved model of “Human Information Processing and Decision-Making” published by [46].

With the rapid technological changes, in many cases, information, communication and mental load became potential problems that required aviation systems to monitor and control. These load systems, therefore, were defined separately in the new model (Figure 4). By this way, operator load is divided into five groups, namely work, task, information communication, and mental load which called as operator total load. The operator total load monitoring and management systems are created and tested in a flight simulator.

(iii) Information Processing Model: In order to understand how to monitor and measure a total load of an operator, it is crucial to have a clear understanding of how operators acquire and analyse information for “decision-making” and “performance action”. Various studies in the literature try to model human information processing systems [44,45]. One of the most recognised and clear explanations of the human information processing model was given by Wickens [46]. This information model was framed around five key components: (i) initial sensors (eyes, ears), (ii) perception, (iii) human memory (working and long-term memory), (iv) decision and response selection, and (v) response and execution. According to this model (Figure 5), the starting point of the information process is the initial sensors, namely eyes and ears. The human brain then processes the detected information. Working memory is a short-term (recent) memory that maintains some amount of information in the mind to enable its manipulation for further information processing while long-term memory is used for storing information and knowledge. The processed incoming information can be temporarily stored and manipulated in the working memory for supporting the human decision-making process. This stage can be described as “main thinking” and is connected with long-term memory where information and knowledge can be stored for more extended periods of time. The most appropriate response, finally, can be executed and accordingly, the decision can be made in the last stage.

The information model of Wickens [46] was improved and adapted to operators by including (i) sensory memory, (ii) situation awareness, (iii) skill and competence, and (iv) load measuring techniques (Figure 5). According to the improved model, after receiving information by the operator senses, receptors encode stimuli from the external environment. Thereafter, the collected information might be transmitted through the sensory memory which is limited a certain amount of information that can be processed for a concise time, about half a second to three seconds, and forwards to working memory. Finally, information might be encoded and stored in long-term memory.

This information process highly depends on the operator’s skill, competence, experience, physical and physiological condition. Moreover, the total load of operators can be monitored from their responses during “situation awareness”, “decision-making” and “performance actions”. The information processing is linked with the reaction time of operators. Due to highly automated systems, information load increases during some phases of flight such as take-off, approach, and landing particularly. It is, therefore, these flight phases that can generate a high mental state which can, in turn, lead to increased “reaction time”, and reduced “decision-making time”.

According to Cummings [47], a person is capable of processing three bits of information per second on average without error. In cases of an operator receiving higher than three bits per second, the occurrence of unavoidable errors and loss of information can be expected. However, the rate of information processing highly depends on the operator’s characteristics such as operator skill, competence, experience, total load, physical and physiological condition.

### 2.3. Sensor Integration into the Working Environment and Experiment Details

Human behaviour, such as the decision-making process, reaction time, and hesitation frequency, is one of the hot recent research areas of the mind in the fields of neurophysiology and human factors, because of its strong connection with the operator’s performance variation, and how it affects human errors. Advances in sensor and data integration technologies in the current transport systems, allow researchers to collect, measure, monitor, and evaluate data prospectively to cope with cognitive, educational and operation challenges, such as maintaining operator load systems, re-designing training and reducing economic consequences [48,49,50]. The modern sensors are out-of-the-box technology and their costs are significantly high. However, with the continuous development of the technology, the cost of the sensors has been dropping and will continue its downward trend over the next years. In addition to this, sensors become smaller in size and require less energy to function. The newly developed sensor systems will let operators and supervisors better understand how operators can cope with their loads while having multiple tasks. In this sense, even though the cost of the ultimate technologies is expensive, using this technology has beneficial outcomes both in the economy by reducing the cost associated with maintenance, fuel spends, time-saving, and as well as saving human life by eliminating human errors, particularly in abnormal and emergency situations.

These new and emerging technologies enable the creation of new microsensors that might be integrated into the operators’ working environment, namely into the driver and pilot cockpits, and ATCOs’ working room (not only integrated into the table and chairs).

The sensors can be classified as follows:

- passive—built into the environment for a distance from the operators, like eye tracking, infrared and cameras, etc.,
- passive-integrated—sensors integrated into the working environment for direct sensing of the operators’ behaviours (such as heart rate, and skin resistance, etc.) built into the operators’ control elements or their clothes,
- semi-active sensors—measuring some characteristics such as the reactions of operators on some signals, and information, etc.,
- active—such sensors that measure the reactions of the operators in response to the specially generated signals (including signals initiated for testing the system and operators).

In this research, several load measuring techniques were created and applied by physiological measures such as video cameras, electrodermal activity (EDA) Open Source Bio-Monitor (OBIMON) devices, eye-tracking devices and outside measuring equipment in the flight simulator and ATC/ATM simulation laboratory of the VRHT at BME.

(i) Participants

The concept was tested in the flight simulator of a Piper Seneca 3 multi-engine aircraft with former student pilots of VRHT with limited experience, and experienced flight instructors working at the department of VRHT. No accidental events were reported by the participants.

(ii) Experiment Design

In this research, three flight scenarios were created: Visual Meteorological Conditions (VMC), Instrument Meteorological Conditions (IMC), and IMC with Attitude Directional Indicator (ADI) failure.

Scenario 1: Visual Meteorological Conditions (VMC): This is an aviation flight category in which visual flight rules apply; expressed in terms of visibility, ceiling height, and aircraft clearance from clouds along the path of flight. In this category, pilots have sufficient visibility to control the aircraft by mostly looking out the window. It is entirely the pilot’s responsibility to navigate the aircraft, maintain visual separation from terrain and other aircraft during operation, and find an airport to safely land.
Scenario 2: Instrument Meteorological Conditions (IMC): This is an aviation flight category that requires pilots to conduct most of the operation by looking at the flight instruments on the control panel rather than by looking out the window under visual flight rules (VFR). This category requires pilots to have more flying skills, tacit knowledge and experience.
Scenario 3: IMC with Attitude Directional Indicator (ADI) failure: In this scenario, pilots control the aircraft under Instrument Meteorological Condition (IMC) with Attitude Directional Indicator failure. This scenario is the most difficult task for a pilot to carry out.

(iii) Task Protocol

The participant pilots are lined up and holding short at RWY 25L (Runway 25Left). They configure the aircraft for take-off (trim +5, flaps take-off, fuel pumps on, landing light on) and check the instruments.

Take-off from RWY 25L

- Set power for take-off (Manifold pressure 40)
- Airspeed (blue line speed)
- Set 300 feet AGL-Above Ground Level gear up flaps up, set climb power (manifold pressure 39, RPM-Revolutions per min 2600), fuel pumps off, landing lights off
- Maintain runway heading (250°) until the aircraft reaches 1000 feet MSL-Mean Sea Level
- Climbing right turn to heading 340°
- Stop climbing at 2500 feet and turn right to heading 070° for downwind.

Fly straight on downwind

- Maintain 2500 feet
- Configure the aircraft for the cruise (Manifold pressure 22, RPM 2400)

Approach and land on RWY25L

- Reduce power for approach (RPM forward, throttle pull back as required), fuel pump on, landing lights on
- Turn right to HDG-Heading 160°
- Set approach flaps and gear down when airspeed is in the white arc.
- Approach speed: blue line speed
- Land on RWY 25L
- Stop on the runway

Use of Integrated Microsensors: Integrated microsensors can be used for improving the operators’ working environment to monitor and manage the total load system. In order to measure the mental state of operators and become familiar with operator behaviour, several microsensors were integrated into a side-stick and a computer mouse by [51,52]. These integrated devices consist of a heartbeat counter sensor, skin conductance, skin temperature, and strain gauges to measure grasp force applied by operators on the handle (how hard operator holds a side-stick or computer mouse) (Table 1, number 2). The heart rate measurement is based on photoplethysmography (PPG) using infrared photo LED-Light Emitting Diode and phototransistor placed next to each other in the handle under the pilot’s index finger. The skin conductance sensor measures how sweaty the pilots’ palm is while the temperature sensor provides information about the pilots’ skin temperature. In the flight simulator laboratory of VRHT at BME, a series of tests were performed to measure the characteristics of pilots with different skills. New computer software was developed by [53,54] to log data coming from the sensors, flight parameters, and the reaction time of the operators. Different operators with different skills and flight experience were tested in many flight situations with different stress levels to characterize the operators.

Electrodermal Activity Device (EDA): Electrodermal activity (EDA) is the property of the human body that causes continuous variation in the electrical characteristics of the skin. It is a psychophysiological indicator of emotional arousal generated by the sweat glands. Mental stress, respiration, and psychological changes are the primary emotional activators that cause strong reactions in the human body. With the activation of any of these factors, the human brain sends signals to the skin to increase the level of sweating. As a result, human skin reacts and becomes a better conductor of electricity. Electrodermal Activity (EDA) has been one of the most widely used methods over 200 years [55]. EDA can be measured from the skin surface by applying an electrical potential between two points. The amount of sweat glands varies across the human body but is the highest in the palmar surface, wrist area, shoulders, forehead and soles of feet regions [56]. These places of the human body have the highest density of eccrine sweat glands that respond to the emotional stimuli. EDA devices, therefore, should be placed on these body parts where changes in sweat gland activity can be easily detected. The activity of sweat glands causes skin conductance and sweating causes a brief drop in the electrical resistance of the skin.

In collaboration with the Department of Affective Psychology at Eötvös Loránd University (ELTE), EDA measurement was realised in the flight simulator of the VRHT at BME (Table 1, number 3). For the present study, the skin conductance activity of an experienced pilot was measured with the Open Source Bio-Monitor (OBIMON) for electrodermal activity in a flight simulator. OBIMON is a small and reliable device capable of synchronised measurement that was used to record EDA from the wrists and shoulders of a pilot (Table 1, number 3). It measures sweat gland activity by taking into account the “Skin Conductance Level” (SCL).

Use of Video/Motion Cameras in the Flight Simulator: Video and motion cameras can be used for measuring the eye movement, and eye blink rate of operators in a complex environment. Given the importance of eye movements for visual perception, there has been a surge of interest in eye movements with numerous studies being conducted to clarify what kind of information can be derived from eye movements. A number of studies have suggested that eye movement, blinking rate, and fixation duration can be linked to the task, information processing, stress level, fatigue and loads [57,58,59]. Many investigators have reported that an increase in eye movement when the task increases in difficulty [60,61]. Rui Fu et al. [60] reported that as the complexity of the task increases, an operators mental load increases, which leads to an increase in eye movement of operators. However, some investigators have reported otherwise; they found an inverse relationship, a decrease in eye movement with increasing task difficulty. For example, May et al. [62] indicated that eye movements were restricted as counting complexity increased. When the level of task difficulty increases, the total load of operators is also increasing, mainly work, task and mental load. There were two motion cameras installed in the flight simulator cockpit by the current researchers. The main aim of the investigation was to demonstrate the difference in eye motion changes through different flight scenarios and to compare the differences between the experienced pilot and less-skilled pilot.

In this experiment, the number of eye movements was counted for an experienced and less-skilled pilot. In addition to this, in order to examine the number of eye movements and eye blink rate during different flight tasks, three flight scenarios were designed: (i) Visual Meteorological Conditions (VMC), (ii) Instrument Meteorological Conditions (IMC), and (iii) IMC with ADI (Attitude Directional Indicator) failure (Table 1, number 4).

Eye-Tracking Device: Eye movement is a necessary component of the visual analysing of a human being. Eye movements reflect human emotions and stress, and cannot be hidden, unlike other emotional demonstrations such as hand movements or voice changes. This speciality, therefore, lets researchers observe eye movement in relation to many scientific studies, including psychology, human behaviour, cognitive science, marketing, and applied research fields. The eye-tracking technology was used to track the eye movements by recording fixations and saccades within the time of the test. The eye-tracker records and saves the information that can be presented with a heat map, cluster, and gaze plots. Eye movements provide numerous clues to underlying cognitive processes as operators encode information, and what information they use or ignore related to their performance under flight scenarios. It has been gaining popularity around for over a hundred years [63]. Amongst research that has been carried out for developing eye-tracking systems, there exists research in different disciplines such as reading [64,65], human-computer interaction [66,67,68], psychoanalysis [69,70], and overlearned tasks [71]. In aeronautics, the eye-tracking method is used in the context of performance [72,73], visual scanning behaviour [74,75], workload assessment [76,77], cognitive process [78,79], aircraft design [80,81], aircraft maintenance [82,83] and physiological factors (stress, fatigue, drowsiness) [42,84].

In the framework of this research, a TOBII eye-tracker was used to record the visual patterns of the pilots in the flight simulator of a Piper Seneca 3 multi-engine aircraft through failure scenarios, such as engine failure and equipment failure at the Department of VRHT at BME (Table 1, number 1) and the results published in [42].

Heart Rate Measurement: The heart is an active, hollow muscular organ about the size of a clenched fist and weighs about 310 g. It acts as a pump that provides a constant flow of blood throughout the human body. Heart rate refers to how many times the heart contracts and releases in a unit of time, usually per minute (bpm), and it is directly related to the workload being placed on the heart. Heart rate is controlled by the Automatic (involuntary) Nervous System (ANS), which is a part of the Central Nervous System (CNS). ANS uses two branches; the Sympathetic Nervous System (SNS) and the Parasympathetic Nervous System (PNS). The sympathetic nervous system releases hormones to accelerate the heart rate, such as in stress situations [85]. According to the National Emergency Medical Association, the normal heart rate for a human adult ranges from 60 to 90 beats per minute (bpm) at rest. Several factors influence heart rate such as age, gender, weight, exercise, body temperature, emotional state, and stress, etc. [86]. Researchers have proven that stress can cause changes to vital health parameters of a person such as an increase in heart rate, respiration rate, and blood pressure. The monitoring and analysing of the heart rate of operators are one of the most promising measures in mental load detection.

With the integration of heart rate monitor into the operator working environment, the mental load can be measured, monitored and managed in real-time. The physiological parameters of operators can be collected with the help of such medical methods such as Heart Rate—HR, Heart Rate Variability—HRV [42,87,88], Electrocardiogram—ECG [86,89,90,91], Electroencephalography—EEG [89,92,93], and Electromyography—EMG [69,94,95]. Heart Rate and Heart Rate Variability of pilots were measured by Mansikka et al. during a real instrument flight rules proficiency test in an F/A-18 simulator as measures of pilot mental workload [88]. Professor Szabo Sandor Andras evaluated the stress reaction of the heart-brain axis by Heart Rate Variability parameters produced by Firstbeat Bodyguard 2 and adapted to real flight [87,96]. In addition to this, QRS Mid-frequency and R-R interval studies let researchers draw the stress level of operators in a complex environment. For example, a very clear relationship between the complexity of the task and observable mental effort was found by Professor Lajos Izso [66,67,68], that when the observable mental effort is high, the Mid-Frequency Power (MFP) is systematically low.

In this study, heart rate of the pilots was recorded through three flight scenarios (i) Visual Meteorological Conditions (VMC), (ii) Instrument Meteorological Conditions (IMC) and (iii) IMC with ADI (Attitude Directional Indicator) failure (Table 1, number 2–3) and the results were published in [42].

**Table 1:** Possible sensors and their application.

No	Name	Performance	Possible Application	Integration into Working Environment
**1**	TOBII Eye-Tracker	Area of interest, Gaze points, Eye movement Fixation duration	Psychology research (the mental state of operators), Level of operator loads, Human behaviour, Attention level, Performance at the cognitive level	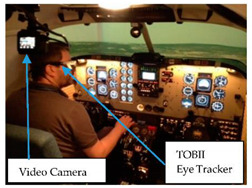 TOBII Eye-Tracker Usage
**2**	Skin Resistance Sensor (a) kin Temperature Sensor (b) Heart Rate Sensor (c)	The resistance of skin (a), Temperature of skin (b), Heart rate (c)	Physiological indicators, Stress level, Emotional state, Mental state, Task difficulty	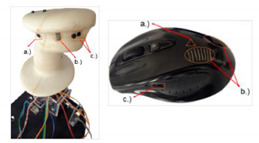 Integrated sensors [53]
**3**	OBIMON (Open Source Bio-Monitor) Electrodermal Activity Device (EDA)	Sweat gland activity by taking into account the “Skin Conductance Level” (SCL)	Arousal level, Stress level, Anxiety level, Mental state, Task difficulty, Operator loads	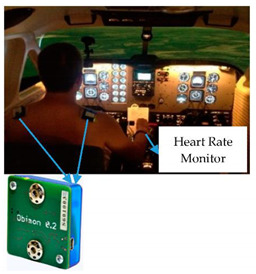 EDA-OBIMON [55] device usage in the flight simulator environment
**4**	Microsensor, Motion and Video Cameras	Eyeblink, Eye movement	Behaviour, Attention level, Performance at the cognitive level, Task difficulty	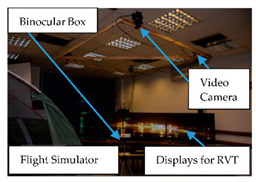 Video camera usage in the simulator laboratory
**5**	Binocular	Providing a magnified stereoscopic view of distant objects, and improve visual acuity	Level of operator loads, Task difficulty	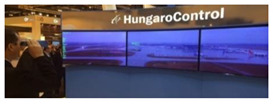 Binocular Usage at HungaroControl [97]
**6**	Load - Tracking Seat Cushion	Fatigue level	Reaction time, Behaviour, Attention level, Performance at the cognitive level, Level of operator loads	Load-Tracking Seat Cushion Usage 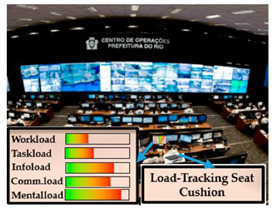 Rio Operation Center Contol Room [98]
**7**	Functional Near-Infrared (fNIR) Spectroscopy	Monitor cerebral hemodynamic changes within the prefrontal cortex in response to the sensory, motor, or cognitive activation.	Mental load assessment in a complex work environment	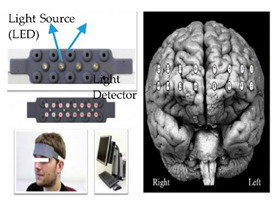 fNIR sensors (right) [99]
**8**	Electrocardiogram-ECG (a), Electroencephalography-EEG (b) Electromyography (EMG) (c) Event-Related Brain Potentials (ERPs) (d)	The electrical activity of the heartbeat (a), Electrical impulses of the brain (b), Electrical activity of nerves and muscles (c, d),	Level of operator loads, Task difficultly, Psychological state, Info. Processing, Emotional level, Cognitive processes in the brain	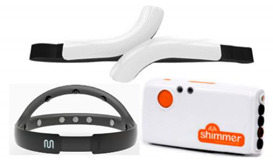 Wearable ECG [100] (top), EEG [101] (left-bottom) and EMG [102] (right-bottom) Monitors
**9**	Breathing-Respiration Sensor	Respiration rate Respiration volume	Mental state, Stress level, Load assessment	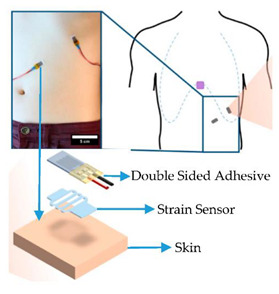 Respiration Sensor [103]
**10**	Force Myography (FMG) and Muscle Strength Sensors	Position and movement of limbs Muscle movement	Stress level, Operator load assessment	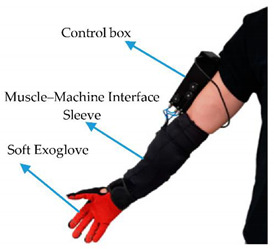 The muscle–machine interface consists of a soft, wearable sleeve that accommodates multiple Force Myography (FMG) and Electromyography sensors [104].
**11**	Blood Oxygen Saturation Sensor	Pulse rate and pulse percentage of haemoglobin in the blood that is saturated with oxygen	Mental state, Stress level, Task difficulty, Operator load assessment	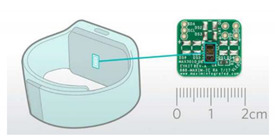 Pulse Oximeter and Heart Rate Monitors [105]

### 2.4. Data Processing

**Figure 6:**
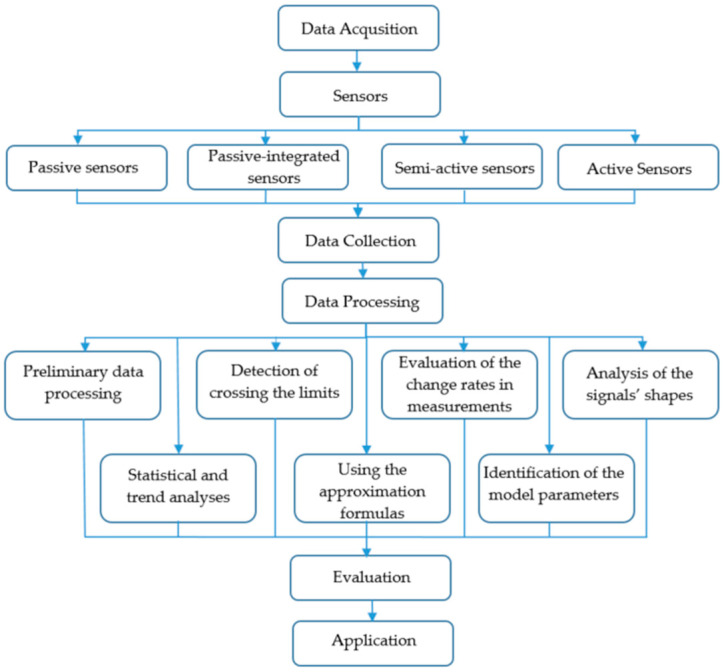
Possible use of the data processing method.

The overall objective of data processing of the measurements is to get the maximum information on the operators’ behaviours, and loads. The following are the major methods that might be used (Figure 6):

- preliminary data processing—filtration, reducing the effects of measured noise,
- detection of crossing the limits—generating warning signals based on crossing the limits by the measured signals,
- evaluation of the change rates in measurements—with detection of the crossing the limits for change rates,
- analysis of the signals’ shapes—including estimation of the maximum deviation, frequency of changing processes, comparison with the standard processes of changing (including changing according to the test signals in active measurements, and active monitoring),
- statistical and trend analyses—estimating the statistical characteristics and parameters of the trends,
- using the approximation formulas—developed especially for these purposes,
- identification of the model parameters—like the transition function of the operators.

The sensors of all the vital health parameters can be built in a compact wearable device (Heart Rate—HR, Heart Rate Variability—HRV, Electrocardiogram—ECG, Electroencephalography—EEG, Electromyography—EMG, Event-Related Brain Potentials—ERPs, Electrodermal Activity—EDA, Functional Near-Infrared Spectroscopy—fNIR, skin temperature, and breathing–respiration sensor, etc.), attached to operator clothes e.g., shirt and gloves (Force Myography—FMG, and Blood Oxygen Saturation Sensor, etc.), and integrated into operator working environment e.g., driver cockpit, tower, or simulator (eye-tracker, infrared thermal camera, and load tracking seat cushion, etc.).

**Figure 7:**
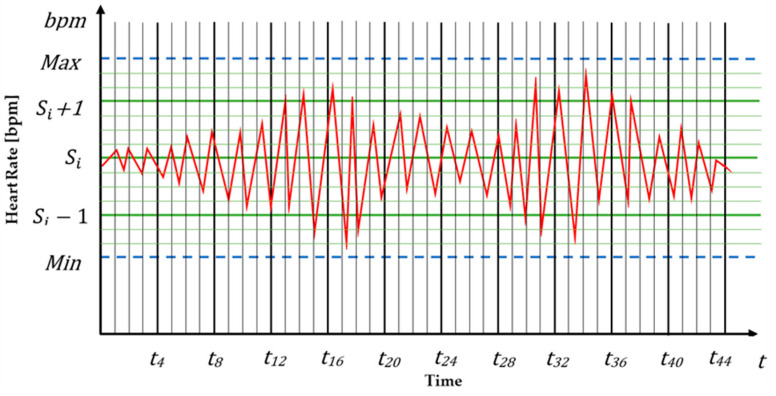
Analysis of operator heart rate measurement.

In the newly built concept, the autonomous system recognizes the operator in the loop and sets all the parameters according to the subject. Then, all the measurable vital health parameters of the operator will be continuously monitored, managed and stored in real-time. The upper (max.) and lower (min.) values of each health parameter would be automatically given by the autonomous system depending on the subject (for example heart rate measurement, Figure 7). The operator recognition system is necessary because vital health signs vary substantially from subject to subject. The processed data of the measured parameters can support operators by giving suggestions, alerting warning signals, and creating situation, fatigue and stress awareness. The autonomous system will identify what is the normal or abnormal value for each of the vital health parameters for a specific subject from the continuously stored data.

In the case of the system detecting any sharp changes in the operator’ vital health parameter(s), unbalanced loads (overload or underload situation) or any confused, stressed or aroused situation, the developed system first generates some suggestions to the operators which will be shown on displays and send alerts/warning messages to the managers and supervisors. Moreover, this concept will let supervisors better understand how operators can cope with their loads while having multiple tasks. If an operator in the loop is incapable of dealing with the routine tasks or in case of a failure, the ground operators (vehicle operation centre, aircraft remote control centres, ATCOs, robot pilots on the ground) will able to take over the control. This innovative device would be very useful in balancing operator over/under loads and decreasing operator errors, thereby reducing the number of incidents and accidents. This system will create more effective interaction between operators and transport systems as well.

### 2.5. Operator Load Monitoring Concept

**Figure 8:**
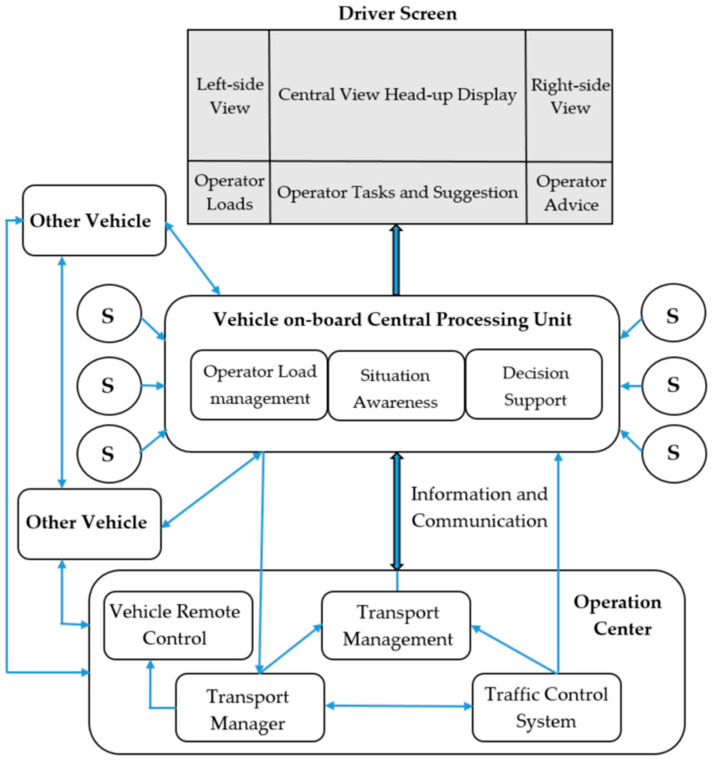
Functional model of the driver decision support system (s—sensors).

The new operator load monitoring concept was developed for drivers to improve the working environment and decision support system. This concept was designed to assist operators with necessary control information and avoiding a crash [106,107,108,109,110]. In this concept, the decision support system of drivers has three layers: (i) operation centre, (ii) vehicle on-board central processing unit, and (iii) smart vehicle screen. The operation centre includes transport management, vehicle remote control, traffic control system and transport manager. The vehicle on-board central processing unit contains (i) operator load management, (ii) situation awareness, and (iii) decision support (Figure 8). The vehicle on-board central processing unit collects and analyses the available data, including the information provided by cooperating with other vehicle and operation centres. With this concept, the driver of a vehicle will see all the necessary controlling information on his cockpit screen. In addition to this, the total load of a driver will be estimated by measuring the driver’s vital health parameters (heart rate, respiration rate, eye movement, skin resistivity, and skin conductivity etc.), human aspects (behaviours, skills, tacit knowledge, experience, physical and psychophysical condition) and external effects (traffic regulation, traffic congestion, and weather condition etc.) by using the integrated sensors and cameras in the vehicle on-board central processing unit.

In case a driver is stressed, aroused, confused or fails to perform routine control actions, the vehicle, first, will generate some suggestions and advice to the driver, and then the operation centre will be immediately informed about the current situation. If the driver still cannot cope with the problem(s), then the operator centre might take over control of the vehicle from the driver. According to the practice of the current researchers, most of the operators understand and are aware of the importance of ongoing technological developments and the desired positive outcomes, that the developed system not only makes their tasks easier and improves their comfort during operation, but also can save human lives by avoiding accidents and incidents. However, on the other hand, there is some minor number of end-users (particularly ATCOs) who are worried that the total load measurement will be used for evaluating their quality of work and performance. It is, therefore, the outcomes of the developed systems were implicitly explained to the operators before the measurements were taken.

### 2.6. Operator Load Management Concept

**Figure 9:**
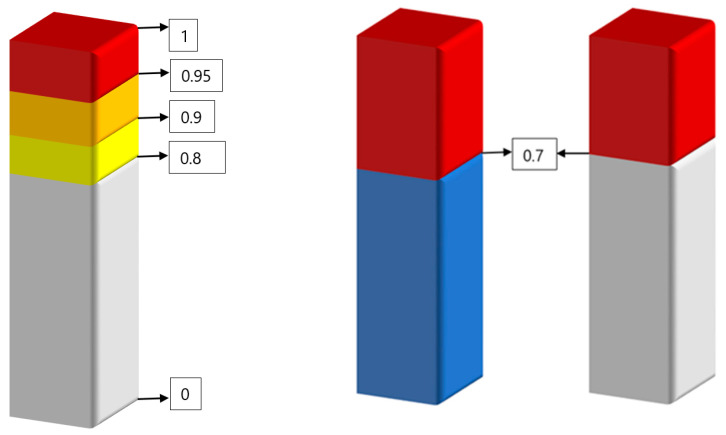
Demonstration of the developed rectangular gauge on a 0–1 scale for overload situation management (left), and combination of operator loads for overload situation management (right).

The operator load management concept was developed separately for overload and underload situations. In the case of the overload situation, two different variations of load management methods can be used; (i) assigning a scoring method—say as in [0,1]—to all the measurements, and (ii) mathematical modelling (Figure 9). According to the assigned a scoring method, all the measurements should be transferred to scores on a 0–1 scale for each operator load where each element should have a weighting coefficient corresponding to different environmental conditions, abnormal situations and failures. According to the developed model, the score is “0” if there is no load, and it is “1” when there is the relative maximum load which can be managed by operators without causing any accidents and incidents. This load can be calculated by actual load over maximum load. There will be five rectangular gauges, each of which displays a different level of operator load currently being experienced by an operator. Each case can generate a weighting coefficient between 0 to 1, and if there is more than one situation that plays a role at the same time, the total score will be the sum of all weighting coefficients.

According to the mathematical model, two different rules might be used for overload situation:

- If one of the operator’s load reaches the threshold where the score is: (i) 0.8—a warning signal must be generated, (ii) 0.9—calling special attention to continuously monitoring the operating condition, and (iii) 0,95—immediate actions are required (Figure 9, left).
- Combination of at least two loads, namely in a case when any two types of load coefficients, reach to 0.7 or above (Figure 9, right): (i) 0.7—warning, (ii) 0.8—monitoring, and 0.9—immediate action required.

**Figure 10:**
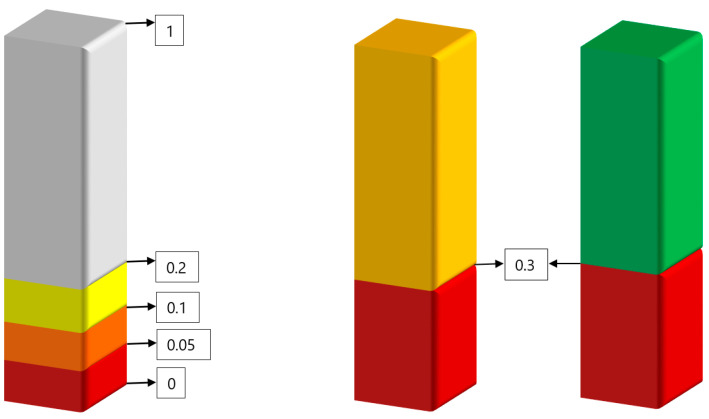
Demonstration of the developed rectangular gauge on a 0–1 scale for underload situation management (left), and combination of operator loads for underload situation management (right).

The operator load management can be developed by the use of more sophisticated methods, like Markov’s decision support. The control field of the vehicle cockpit can be designed by projecting the most necessary information (including loads, tasks, advice) in real-time mode to the vehicle cockpit window instead of having a series of wide screens in control panels. This will help operators to manage their total loads more efficiently and reduce their stress levels at the same time. Another operator load management concept is an underload situation. As early sections indicate, technological developments have shifted the role of the operators from active control to passive monitoring of the automated processes. Unbalanced operator load systems may accompany this highly automated system, and produce unintended reductions in situation awareness, decreases in the quality of decision-making, and increased levels of stress. Sometimes the levels of operator loads become too low where the operator may become inattentive and/or bored. This happens because an operator’s job sometimes can get monotonous. These situations are generally referred to as operator underload, including work, task, information communication and mental underload. Operator underload caused attention to be withdrawn, leading to the decrement. In order to manage operator underload, a management model was developed. According to the mathematical model, two different rules might be used for underload situation (Figure 10):

- If one of the operator loads drops to the threshold where is the score: (i) 0.2—a warning signal must be generated, (ii) 0.1—calling the special attention to continuously monitoring the operating condition, and (iii) 0.05—immediate actions are required (Figure 10, left).
- Combination of at least two loads, namely in a case when any two types of load coefficients drop to 0.3 or below: (i) 0.3—warning, (ii), 0.2—monitoring, and 0.1—immediate actions required (Figure 10, right).

**Figure 11:**
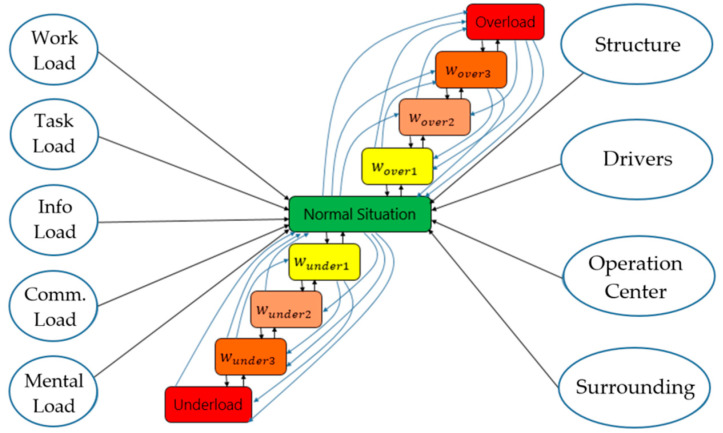
Stochastic load model of flight risk.

In the operator load management model, the space of the load parameter was divided into several sub-spaces as shown in Figure 11, including overload and underload states.

- In the case of the operator overload situations: $w_{over1}=$ 0.8 (a warning signal must be generated), $w_{over2}=$ 0.9 (calling special attention to continuously monitoring the operating condition), and $w_{over3}=$ 0.95 (immediate actions are required).
- In the case of the operator underload situations: $w_{under1}=$ 0.2 (warning signal must be generated), $w_{under2}=$ 0.1 (calling the special attention to continuously monitoring the operating condition), $w_{under3}$ = 0.05 (immediate actions are required).

As seen in Figure 11, the “normal situation” is highly influenced by operator loads (work, task, information, communication and mental load) and as well as several other factors such as structure, surrounding, weather condition, traffic complexity, skill and tacit knowledge of operators, etc. For example, if there is a failure in a transport system or the weather condition is too poor, operators tend to get more nervous than in the normal situation. Figure 9 and Figure 10 describe the situation for each type of the loads, however, operators have the combination of the loads (Figure 11). In such cases, the operator load index can be defined at each key step of the investigation by using the following formula:

(1) $$i_{Load}=\overset{5}{\underset{i=1}{\sum }}w_{e_{i}}\left(u,z\right)L_{c}$$

where $i_{Load}$ is total load index, $w_{e_{i}}$ is weighting coefficient and $L_{c_{i}}$ is the load coefficient, **u** is the control and **z** is the environmental characteristics. In the case of the over/under load situation, two different variations of load management methods can be used based on the load coefficient. The total load index can be determined for each [k+1] step by using the following formula:

(2) $$i_{\mathrm{Load}}\left[k+1\right]=\overset{r=9}{\underset{q=1}{\sum }}\left(A\left[k\right]i_{\mathrm{Load}}\left[k\right]+w_{\mathrm{qu}}B\left[k\right]u\left[i_{\mathrm{Load}}\left[k\right]\right]+w_{\mathrm{qz}}F\left[k\right]z\left[k\right]\right)$$

where $u\left[k,i_{\mathrm{Load}}\left[k\right]\right]$ is the management definition, and **z**[k] is the environment. $u_{1}$ = Work load, $u_{2}$ = Task load, $u_{3}$ = Information load, $u_{4}$ = Communication load, $u_{5}$ = Mental load and $z_{1}$ = Structure (such as mechanical failure, malfunction of the automation system or software errors) $,z_{2}$ = Pilots, $z_{3}$ = ATCOs, $z_{4}$ = Surroundings (such as normal or severe weather condition). Matrixes A, B, F are the load transition matrix where the A matrix is the state transition matrix, the B matrix is the control matrix which takes into account the effect of the applied management control on the operator load, and the F matrix is the influence of the changing environment characteristics on the operator load index.

(3) $$A=\left[\begin{array}{ccc} a_{11} & \cdots & a_{19}\\ \vdots & \ddots & \vdots \\ a_{91} & \cdots & a_{99} \end{array}\right]\;\;and\;\;i_{l}=\left[\begin{array}{c} i_{1}\\ i_{2}\\ i_{3}\\ i_{4}\\ i_{5}\\ i_{6}\\ i_{7}\\ i_{8}\\ i_{9} \end{array}\right]$$

(4) $$B=\left[\begin{array}{ccc} b_{11} & \cdots & b_{15}\\ \vdots & \ddots & \vdots \\ b_{91} & \cdots & b_{95} \end{array}\right]\;\;and\;\;u=\left[\begin{array}{c} u_{1}\\ u_{2}\\ u_{3}\\ u_{4}\\ u_{5} \end{array}\right]$$

(5) $$F=\left[\begin{array}{ccc} f_{11} & \cdots & f_{14}\\ \vdots & \ddots & \vdots \\ f_{91} & \cdots & f_{94} \end{array}\right]\;\;and\;\;z=\left[\begin{array}{c} z_{1}\\ z_{2}\\ z_{3}\\ z_{4} \end{array}\right]$$

## 3. Results

### 3.1. Typical Operator Load Measurements

Due to the wide application of modern technology, operator loads can be monitored and managed by sensors, cameras and innovative medical devices. In this research, such applications like video cameras, electrodermal activity (EDA) OBOMIN devices, and outside measuring equipment were used in the flight simulator and ATC/ATM simulation laboratory of the Department of Aeronautics, Naval Architecture and Railway Vehicles at Budapest University of Technology and Economics [111].

**Figure 12:**
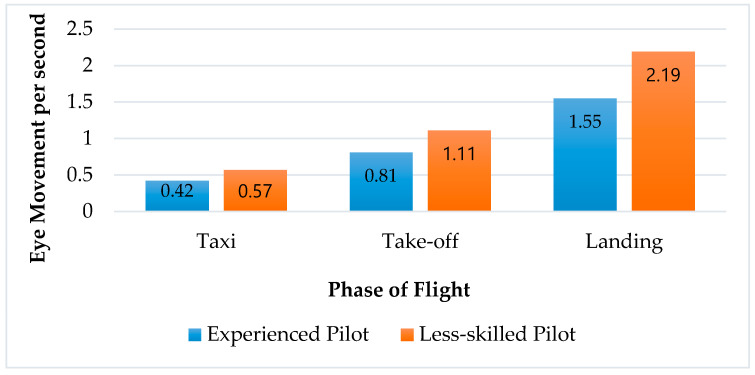
Comparison of eye movement between experienced and less-skilled pilots.

Motion and Video Camera Results: In this experiment, the number of eye movements was counted for the experienced and less-skilled pilots. According to this result, a strong relationship was found between task and operator working behaviours like during taxi, take-off, and landing (experienced and less-skilled pilots) seen in Figure 12. According to the eye movement results, the less-skilled pilots made more eye movements during taxi (35%), take-off (37%) and landing (41%) in comparison to the experienced pilots (Figure 12). On the other hand, in the case of the complexity of task increasing, the number of eye movements per second also accordingly increases.

**Figure 13:**
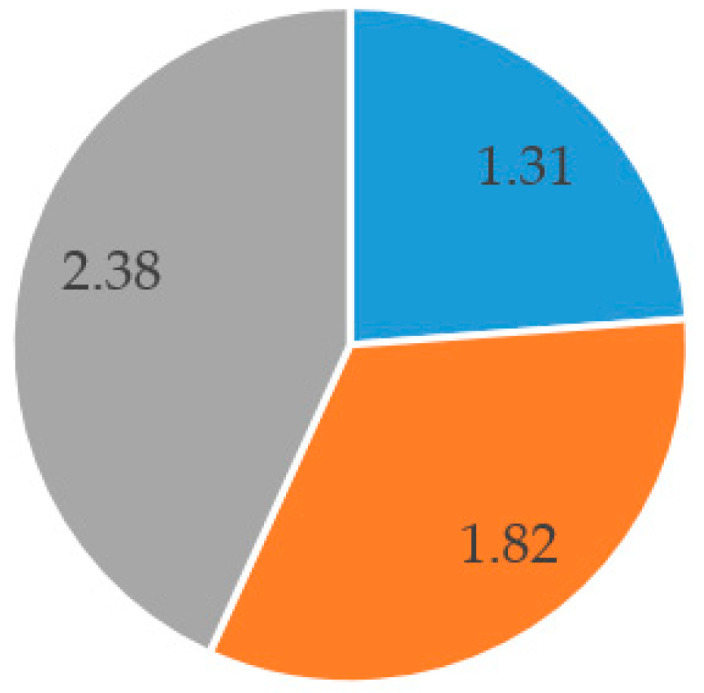
Eye movement comparison of the experienced pilot through different scenarios.

In order to examine the number of eye movements and eye blink rate during different flight tasks, three flight scenarios were designed: (i) Visual Meteorological Conditions (VMC), (ii) Instrument Meteorological Conditions (IMC), and (iii) IMC with ADI (Attitude Directional Indicator) failure and experiment details were given on page number 9. According to the results, the number of eye movements of the experienced pilot was found to (i) 1.31 per second under Visual Meteorological Conditions (VMC) scenario, (ii) 1.82 per second under Instrument Meteorological Conditions (IMC) scenario, and 2.38 per second under IMC with Attitude Directional Indicator (ADI) failure (Figure 13).

**Figure 14:**
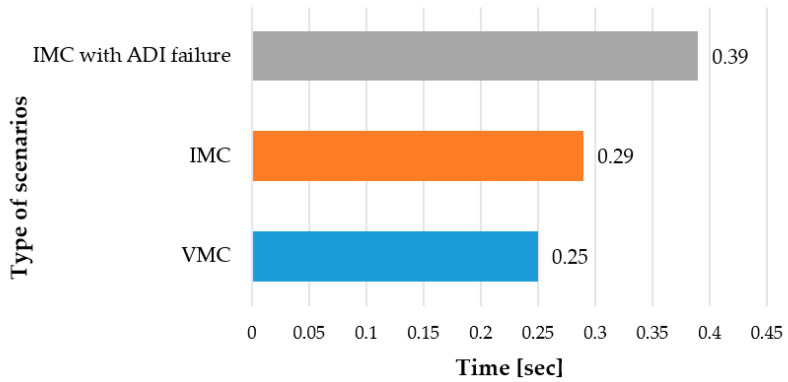
Eyeblink rate of the experienced pilot through different scenarios.

In this research, eye blink rate was used as a measure of studying the connection between the mental state and the complexity of flight tasks. The human eye blinks once every four or five seconds on average—that is approximately 15–20 times per minute [112]. Eye-blink rates can be affected by a variety of different factors such as human behaviour, experience level, task (nature, difficulty, and engagement) and endogenous state (mental activity, psychological state, and state of attention). Several studies have shown that an increased level of task difficulty results in less frequent eye-blinking [113]. According to Jyotsna and Amudha [114], a constant increase in the level of task difficulty will increase the cognitive load, which results in a reduced number of eye blinks. In contrast to these researchers, some other studies reported that the blink rate increases as the task difficulty increases [57,58,60]. Tanaka & Yamaoka studied the relationship between blink rate and task difficulty and reported the more difficult the task became, the higher the blink rate was [57]. Additionally, a limited number of studies found no relationship between the degree of task difficulty and the blink rate. For example, Pauline Cho [115] reported that the level of task difficulty did not affect the blink rate in primary gaze and downward gaze. In this research, the eye blink rate of an experienced pilot was investigated through three flight scenarios. The number of eye blinks (full blink and half blink) of the experienced pilot increased significantly in parallel to the task complexity: (i) 0.25 per second under Visual Meteorological Conditions (VMC) scenario, (ii) 0.29 per second under Instrument Meteorological Conditions (IMC) scenario, and 0.39 per second under IMC with Attitude Directional Indicator (ADI) failure (Figure 14). In addition to this, it was also noticed that eye flutters (rapid muscle movement in the eyebrow area) also increased.

According to the outcomes of the experiments, direct relationships were found between task difficulty and both eye movement and eye blink rate. As discussed earlier, a number of studies support the outcomes and methodology of the experiments. On the other hand, some others do not. However, in the newly built concept, the autonomous system recognizes the operator in the loop, and afterwards, starts to continuously measure and store all the parameters on the subject in the operator environment including eye movement, blink rate, skin resistivity, and heart rate, etc. The autonomous system will identify what is the normal or abnormal value for each of the vital health parameters for a specific subject from the continuously stored data. In the case of the system detecting any sharp changes in the operator’s vital health parameters, the autonomous system will generate suggestions to the operator, and the real-time information will be automatically given to the control managers and supervisors.

**Figure 15:**
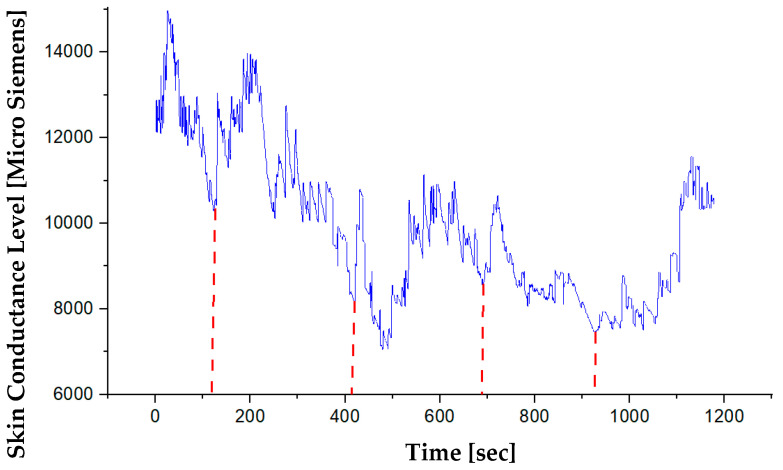
Skin Conductance Level of a pilot during the scenario.

Electrodermal Activity (EDA) Results: In collaboration with the Department of Affective Psychology at Eötvös Loránd University (ELTE), EDA measurement was realised in the flight simulator of the VRHT at BME. For the present study, the skin conductance activity of an experienced pilot was measured with Open Source Bio-Monitor (OBIMON) for electrodermal activity. OBIMON is a small and reliable device capable of synchronised measurement that was used to record EDA from the wrists and shoulders of a pilot (Figure 15). It measures sweat gland activity by taking into account the “Skin Conductance Level” (SCL). For example, when operators are emotionally aroused, sweat glands increase function and skin conductance level increases as well. Skin conductance is an indicator of sweat gland function, so whenever the operator aroused, stressed or unbalanced loaded, sweat glands increase function, and the skin conductance level gets higher accordingly. The Skin Conductance Level of a pilot was recorded during all phases of the flight.

The results suggested that emotional arousal was highest during flight take off in comparison to en-route and landing. Moreover, based on analyses of the measured EDA, the arousal was found to be high when the flight took turns. This is an interesting finding that needs to be replicated in further studies. In addition to this, a TOBII Eye-tracker was used to record the visual patterns of the pilots, and Heart Rate Measurements were also performed through failure scenarios in the flight simulator of the Department of VRHT at BME. The results of the measurements were published in [42]. According to the results, eye-tracking and heart rate can be used to assess the degree of mental status.

### 3.2. Load Monitoring Concepts for Pilots and ATCOs

**Figure 16:**
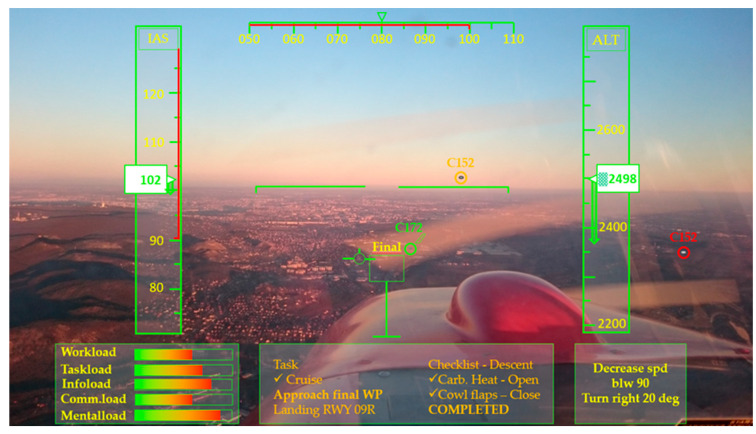
Future load monitoring and management design in the cockpit.

**Figure 17:**
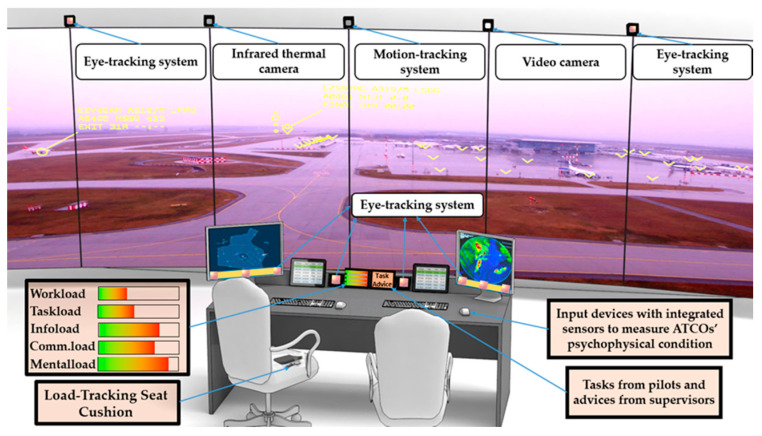
Future load monitoring and management design in Air Traffic Management (ATM).

The operator load monitoring and management concepts were developed separately for pilots (Figure 16) and ATCOs (Figure 17). In the pilot concept, there are five types of operator loads which vary depending on the measured loads that are presented by small colourful columns on the pilot’s screen (Figure 16, left bottom). The second part is the “task zone” which is associated with the air-ground communication and recommendations from pilots and air traffic (Figure 16, middle bottom). Finally, the third part is the “advice zone” in which pilot receives all the necessary advice on the cockpit window from the ground (ATCOs, aircraft remote control centres, robot pilots on the ground) or on-board (robot pilots or other aircrafts’ pilots) (Figure 16, right bottom). For example, with upcoming technologies, the traditional two pilot set-up will be reduced to one on-board and one robot co-pilot on-board or the ground. The advice which will come from these virtual robot pilots can also be shown on the cockpit window.

Concerning the concept of ATCO, there are several load monitoring devices integrated into the ATCO’s working environment such as eye-trackers, heart rate monitors, skin temperature sensors, motion tracking systems, infrared thermal camera systems and load tracking seat cushion sensors, etc. (Figure 17). In this concept, on the one hand, all the physiological parameters of ATCOs will be monitored and stored in real-time, and the five types of loads will be presented accordingly on the ATCO’s screen. On the other hand, there will be a display for tasks and advice, which helps to avoid misunderstanding between operators.

## 4. Discussion

### 4.1. Evaluation of the Results

In highly automated systems, the role of mental condition was found to be increased, and task and workload became more interconnected, and information load and communication load were detected as new types of operator loads. Thereby a new operator load model was created and divided into five categories, namely work, task, information, communication and mental load. Eye movement, visual attention and eye blink of pilots (experienced and less-skilled) were measured in the flight simulation during take-off and final approach. According to the outcomes, in the case of the complexity of task increasing, the number of eye movements, and eye blink accordingly increased. Based on these results, a recommendation of using eye-tracking systems was developed for operator training in the flight simulator. This measurement lets the current researcher draw a mental picture of an operator in real-time. On the other hand, the results of EDA measurement suggested that emotional arousal was highest during flight take-off in comparison to en-route and landing. Moreover, based on the analyses of the measured EDA, the arousal was found to be high when the flight took turns. This measurement shows that the skin conductance level of operators can be measured continuously during their operation; the results let the current researcher monitor the mental load of operators. Lastly, operator load management systems were built for under and overload situations by using this measurement.

With the developed system, operators will have tasks according to their current conditions, including the level of total loads, state of physical and psychological parameters, and other aspects (traffic complexity, weather condition, unlawful actions, etc.). In the new system, the vital health parameters of operators will be continuously measured and stored during operation. In this concept, the autonomous system recognizes the operators in the loop and in the case of the system detecting any unbalanced loads (overload or underload situation), the system first generates some suggestions to the operators which will be shown on their screens, and sends alerts and warning messages to the managers and supervisors. If the operators in the loop are incapable of dealing with the routine tasks (such as strip marking, transferring an aircraft to the next sector) or in the case of failure, the control of the aircraft will able to take over from the pilots in the loop by ground operators or the fatigued ATCOs can be replaced with fresh ones. The overall result of this paper suggested that the developed load monitoring and management methods serve as an excellent tool for balancing the total operator load, thereby improving the performance of operators and increasing the safety of operators’ actions, particularly in an abnormal/emergency situation. The consequences of this development in this research are: (i) monitoring operator total loads, (ii) managing operator actions, (iii) increasing the level of situation awareness, (iv) reducing operator loads on the subject, (v) better decision making and improving the quality of the decision, (vi) increasing operator effectiveness and productivity, and (vii) increasing safety particularly in abnormal/emergency situations. The outcomes of this paper will be useful in balancing operator total load, creating operator training courses, designing operator working environments (cockpit and ATC systems), and decreasing communication errors between operators, etc.

### 4.2. Load Management Concept Validation

Concept verification, validation and testing were continuously used during this research, and as well as model development simulation. Only the major elements are summarized here. The current authors adopted and developed a new concept on operators’ load modelling, monitoring and management that could be used in simulation and testing in simplified forms as simulation and the testing element of the concept. The current researchers, therefore, verified and validated the concept only. Concept validation means the evaluation of the developed operator load models and monitoring and management of the real processes. During the concept validation, the following aspects were studied: (i) before starting the real measurements, the vital health parameters of the invited pilots were measured in the flight simulator, (ii) same pilots and tasks were used for all of the measurements (eye movements, electrodermal activity, and heart rate, etc.), and a test flying was performed through predefined simplified tasks before the measurements were taken.

## 5. Conclusions

Due to rapid development in automated systems in aviation, it is expected that the role of operators will be changed from active controlling to passive monitoring. This highly automated system may be accompanied by unintended reductions in situation awareness, unbalanced operator load, increased stress, and issues of mistrust, boredom, and monotony. While the responsibility of the operator to fly safely remains unchanged, several new skills are required to control the aircraft. The future operator environment (cockpit and future ground control tower for pilots) needs to be redesigned taking into account various psychological parameters, human factors, and total operator load systems. Operators need viable constructs, principles and aviation systems to promote a better understanding of automation and balance their loads in complex circumstances.

The present research had the scope to develop general load monitoring and management systems working in highly automated systems. To do so, first, the role and loads of operators were investigated and analysed by using (i) outside measurements (like motion cameras, infrared thermal cameras), and (ii) connecting directly to the operator’s body (EDA—Electrodermal Activity Device—OBIMON and heart rate monitor). According to the overall results, all the investigated measuring methods fully support the operator load index classification and allow the implementation of load monitoring and display systems for pilots (Figure 16) and ATCOs (Figure 17). Second, some of the well-known operator models like “load model” by Endsley, “Swiss Cheese Model” by James Reason and “information model” by Wickens were improved and adapted to human operator work that enables full modelling of the operator situation awareness and decision processes in highly automated systems (Figure 3, Figure 4 and Figure 5). Third, a new operator load model (namely: work, task, information communication and mental) was created, tested and verified in flight simulators and partially validated in real situations (Figure 4). Fourth, the Formulas (1)–(5) were defined for the load index calculation method of operators. Finally, the total load management rules were built and management methods were developed based on workload, task, information, communication, and mental load for overload and underload situations (Figure 9 and Figure 10).
